# Macroalga-associated bacterial endophyte bioactive secondary metabolites twinning: *Cystoseira myrica* and its associated *Catenococcus thiocycli* QCm as a model

**DOI:** 10.1007/s11274-022-03394-2

**Published:** 2022-08-25

**Authors:** Noura Sh. A. Hagaggi, Usama M. Abdul-Raouf

**Affiliations:** Department of Botany, Faculty of Science, Aswan University, Aswan, 81528 Egypt

**Keywords:** Marine, Macroalgae, Endophyte, Bacteria, Metabolites, Relationship

## Abstract

Marine ecosystems represent the largest biome on the earth. Until now, the relationships between the marine microbial inhabitants and the macroalgal species unclear, and the previous studies are insufficient. So, more research is required to advance our understanding of macroalgal- microbial interactions. In this study, we tried to investigate the relationship between the brown marine macroalga, *Cystoseira myrica* and its associated bacterial endophyte, *Catenococcus thiocycli*, as the first study concerning the production of bioactive secondary metabolites from a macroalgal species comparing with its associated endophytic bacteria. Secondary metabolites were extracted from alga and its bacterial endophyte with ethyl acetate and methanol. All extracts contained significant quantities of phenolics, flavonoids, tannins, and saponins. Strikingly, extracts possess antioxidant, anti-inflammatory and antimicrobial activities which were significantly correlated to phenolic and flavonoid contents.

## Introduction

Marine organisms that survive in extreme conditions possess potential to produce unique compounds that are not present in the terrestrial organisms. Over the past two decades, searching for bioactive natural products from the marine environment is of great interest to scientists due to their broad pharmaceutical benefits (Madkour et al. 2019).

Marine endophytic bacteria are living within the inner tissues of marine plants without causing any harmful effect to the host. Among the various marine organisms, macroalgae (seaweeds) that have wide diverse of bioactive compounds including flavonoids, terpenoids, alkaloids, quinones, sterols, tannins and polysaccharides are abundant sources of marine endophytic bacteria that attracting interest of biochemical research (Erbabley and Junianto 2020).

Although several studies focused on the relationships between epiphytic bacteria and their macroalgal hosts, reports about endophytic bacteria from macroalgae are still insufficient (Ameen et al. 2021). Consequently, there are many gaps in knowledge, and it is necessary to continue searching to achieve a better understanding of the relationship between macroalgae and associated bacteria (Soria-Mercado et al. 2012; Ameen et al. 2021). Due to, many species of marine algae have been reported as potential source for new drugs, the phytochemical screening of all marine algae and their associated microorganisms should become a priority, in order to determine which species can be exploited (Erbabley and Junianto 2020; Rashad and El-Chaghaby 2020). Wherefore, this study was carried out to investigate the relationship between the brown marine macroalga *Cystoseira myrica* (S.G.Gmelin) C. Agardh and the associated endophytic bacteria in regard to the production of bioactive secondary metabolites, in order to involve towards our understanding of macroalgae-bacteria relationships, and for exploration a new marine sources of potential therapeutic and pharmaceutical agents.

## Materials and methods

### Collection of algal material

Samples were collected from Egyptian Red Sea, El Quseir (26°06ʹ14ʹʹN 34°16ʹ52ʹʹE) during April 2021, based on morphological characteristics like brown color, elongated main axis (about 50 cm long) bears spine-like appendages, air vesicles and distichous lateral branches followed the keys and descriptions adapted from Aleem (1993) and the website (http://www.seaweed.ie/descriptions/). Samples were transferred to the laboratory using cooling box for further study.

### Isolation and identification of endophytic bacteria

Samples were carefully surface sterilized using ethyl alcohol (70%) and sterilized distilled water to remove epiphytic microorganisms. Filtered-autoclaved natural seawater, and synthetic seawater agar, contained (g/L: NaCl, 30; Na_2_SO_4_, 4; MgCl_2_.7H_2_O, 1; KCl, 0.7; NH_4_Cl, 0.5; NaHCO_3_, 0.2; KH_2_PO_4_, 0.2; CaCl_2_.2H_2_O, 0.1; KBr, 0.1 and H_3_BO_3_, 0.025) were used for isolation and subculturing. The samples were aseptically crushed in 5 ml sterilized saline solution. 100 μl of tissue extract was spread on seawater agar plates. Plates were incubated at 25 °C for 72 h and observed daily for the appearance of colonies.

The obtained isolate was commercially sent to SolGent Co., Ltd., South Korea for 16S rRNA gene sequencing. Alignment was performed between the obtained sequence and NCBI reference sequence database. The present sequence was introduced into NCBI to obtain an accession number.

### Extraction of secondary metabolites

In Erlenmeyer flasks, 20 g of algal powder was extracted separately with methanol and ethyl acetate at a sample: solvent ratio of 1:5 (*w*/*v*) (Nurjanah et al. 2017). Flasks were tightly covered and shaking at 150 rpm for 24 h at 37 ℃. Extracts were then filtered and solvents were evaporated at the room temperature. The obtained crude extracts were kept in the refrigerator for further study.

Bacterial extraction was carried out by inoculating strain QCm in 5 L nutrient broth and incubating at 37 °C under 150 rpm for 72 h. Then, the supernatant and the cell mass were extracted using methanol and ethyl acetate according to Noha et al. (2013).

### Estimation of secondary metabolites contents

Stock concentrations of 1 mg/mL of algal and bacterial extracts were prepared. The contents of active secondary metabolites i.e., phenols, flavonoids, saponins and tannins were estimated in each extract according to Visweswari et al. (2013).

### Assessment of in vitro antioxidant activity

#### Total antioxidant activity

The total antioxidant activity was estimated as µg ascorbic acid equivalents/mg extract using phosphomolybdenum assay (Prieto et al. 1999).

#### 2,2-diphenyl-1-picrylhydrazyl (DPPH) radical scavenging activity

The ability of the extracts to scavenge the stable free radical DPPH was measured according to the method of Brand-Williams et al. (1995). In brief, 2 mL of the DPPH solution (0.5 mmol/L) was added to 1 mL of the tested extract and incubated in darkness for 30 min at room temperature. After that, the absorbance was measured at 517 nm against control. The percentage of free radical scavenging was calculated as the following:

$$ {\text{DPPH}}\,{\text{ radical }}\,{\text{scavenging }}\left( \% \right)\, \, = \, \, \left[ {\left( {{\text{A}}_{{{\text{Control}}}} {-}{\text{ A}}_{{{\text{Sample}}}} } \right)/{\text{A}}_{{{\text{Control}}}} } \right] \, \, \times \, \,{1}00 $$

#### Ferric ion reducing power

The method of Ganesan and Kumar (2008) was followed to evaluate the reducing power of the extracts. Briefly, an aliquot of 50 μL extract was added to 0.1 mL of 1% potassium ferricyanide. The mixture was incubated at 50 °C for 30 min. Then, 0.1 mL of 1% trichloroacetic acid and 0.1 mL of 0.1% FeCl_3_ were added to the mixture and left for 20 min. Absorbance was read at 700 nm. The reducing power of the extracts was calculated using ascorbic acid standard curve (Yen and Chen 1995).

#### Hydrogen peroxide (H_2_O_2_) radical scavenging activity

The ability of extracts to scavenge H_2_O_2_ radicals was estimated according to Ruch et al. (1989). 0.1 mL of each extract was transferred into the test tubes and was made up to 0.4 mL with phosphate buffer. After that, 0.6 mL of H_2_O_2_ solution (40 mM) was added and vortexed. The absorbance of the mixture was read at 230 nm against control. The percentage of H_2_O_2_ scavenging was calculated as follows:

$$ {\text{H}}_{{2}} {\text{O}}_{{2}} {\text{scavenging activity }}\left( \% \right) \, \, = \, \, \left[ {\left( {{\text{A}}_{{{\text{Control}}}} - {\text{A}}_{{{\text{Sample}}}} )/{\text{ A}}_{{{\text{Control}}}} } \right)} \right] $$

### Assessment of in vitro anti-inflammatory activity

#### Inhibition of protein denaturation

The ability of the extracts to inhibit protein denaturation was determined using the method of Mizushima and Kobayashi (1968) with slight modifications. The reaction mixture which consisted of 1 mL of 1% aqueous solution of bovine serum albumin and 1 mL of the tested extract was adjusted to pH 6.3 using 1 N HCl, incubated for 20 min at 37 °C and then heated to 50 °C for 30 min. After cooling to room temperature, the turbidity of the sample was measured at 660 nm. Aspirin (100 μg/mL) was used as standard anti-inflammatory drug. The percentage inhibition of protein denaturation was calculated using the following equation:

$$ {\text{Percentage}}\,{\text{ inhibition }}\left( \% \right) \, \, = \, \, \left( {{\text{A}}_{{{\text{Control}}}} - {\text{A}}_{{{\text{Sample}}}} } \right) \, /{\text{A}}_{{{\text{Control}}}} \, \times \,{ 1}00 $$

#### Anti-proteinase activity

The Anti-proteinase activity of the extracts was estimated according to Oyedapo and Famurewa (2008). 1 mL of the tested extract was added into the reaction mixture which contained 1 mL of (20 mM) Tris HCl buffer (pH 7.4) and 1 mL of (0.06 mg/mL) trypsin. The mixture was incubated for 5 min at 37 ℃. Then, 1 mL of (0.7%, *w*/*v*) casein was added and incubated for 20 min at 37 °C. After that, the reaction was stopped by 2 mL of (70%, *w*/*v*) tricholoroacetic acid. The reaction mixture was centrifuged, and the absorbance of the obtained supernatant was measured at 210 nm. The percentage inhibition of proteinase was calculated using the following equation: ${\text{Percentage }}\,{\text{inhibition }}\left( \% \right)\, \, = \, \, \left( {{\text{A}}_{{{\text{Control}}}} - {\text{A}}_{{{\text{Sample}}}} } \right) \, /{\text{A}}_{{{\text{Control}}}} \, \times \, \,{1}00$.

#### Antimicrobial activity

Antibacterial and antifungal activities of the extracts against *Escherichia coli, Salmonella typhi, Klebsiella pneumoniae, Micrococcus luteus, Proteus mirabilis, Aspergillus niger* and *Candida albicans* were investigated using well diffusion method according to the Clinical and Laboratory Standard Institute (2010). Briefly, Mueller Hinton gar plates were inoculated with the tested species. Wells of 6 mm were made using sterilized cork borer. The wells were filled with 50 μL of 1 mg/mL of the tested extract. Plates of bacteria were incubated at 37 °C for 48 h and plates of fungi were incubated at 28 °C for 7 days. Ampicillin and fluconazole (1 mg/mL) were used as antibacterial and antifungal standard drugs respectively.

The diameters of inhibition zones (mm) were recorded. The activity index (AI) was calculated according to Singh et al. (2002) equation:

$$ {\text{Activity }}\,{\text{ index }}\,\left( {{\text{AI}}} \right)\, = \,\frac{{{\text{Inhibition }}\,{\text{zone}}\,{\text{ of }}\,{\text{extract}}}}{{{\text{Inhibition }}\,{\text{zone }}\,{\text{of }}\,{\text{standard}}\,{\text{ drug}}}} $$

#### Gas chromatography-mass spectrometry (GC–MS) analysis

The GC–MS analysis of the extracts was performed in the chemistry department, faculty of science, Aswan university using GC/MS instrument (Agilent Technologies: 7890A GC/5977A MSD). The bioactive compounds were identified by comparing the mass spectrum of the unknown compounds with the spectrum of the known compounds using National Institute Standard and Technology (NIST11.L) mass spectral reference library (Marzoqi et al. 2015).

#### Data analysis

All data were recorded from three biological replicates (n = 3). Data were analyzed using the statistical software R project (v.3.2.2.). All values were represented as means ± standard errors (SEs). Tukey’s HSD- test was used to compare between extracts. The level (*P* ≤ 0.05) was considered as significant. Principal Component Analysis (PCA) was performed to achieve the multivariate analysis of the secondary metabolites in the host macroalga and the associated bacterial endophyte using XLSTAT software (v. 2020.1.3). Pearson’s correlation was run to determine the correlations among the different metabolites and their biological activities.

## Results

### Identification of endophytic bacteria

The comparative analysis of strain QCm 16S rRNA gene sequence with NCBI reference sequence database showed that strain QCm exhibited the highest similarity of 100% with *Catenococcus thiocycli* strain TG 5–3 (NR104870). The present sequence was deposited to NCBI database under the accession number (OK584768).

### Estimation of secondary metabolites

**Fig. 1:**
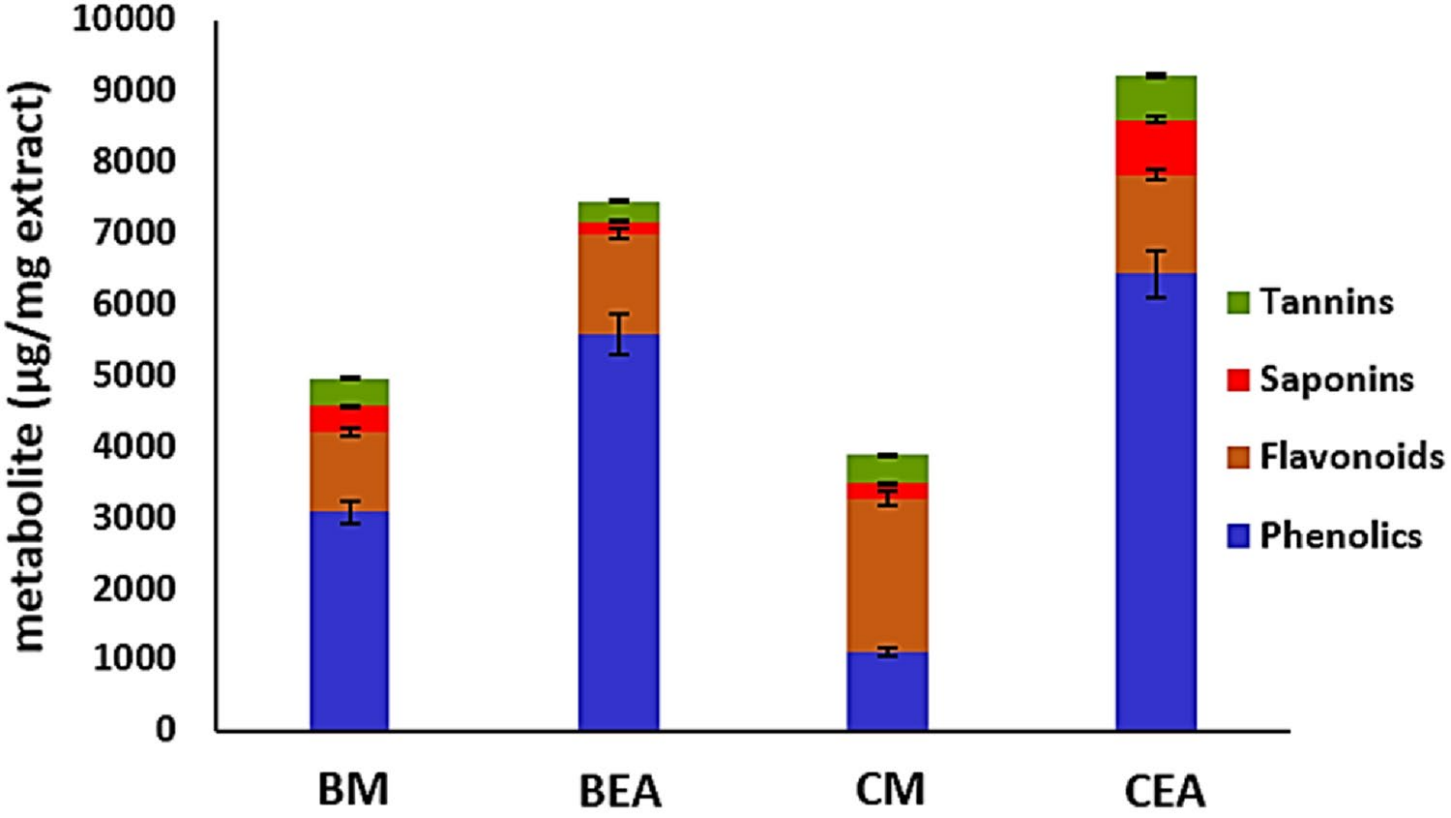
The content of different secondary metabolites in the different extracts of *C. myrica* and its associated endophyte *C. thiocycli*. Extracts: *BEA* *C. thiocycli* ethyl acetate, *BM* *C. thiocycli* methanol, *CEA* *C. myrica* ethyl acetate, *CM* *C. myrica* methanol

The contents of phenolics, flavonoids, saponins and tannins in each of the ethyl acetate and methanol extracts of the endophyte *C. thiocycli* strain QCm and its host alga *C. myrica* were evaluated (Fig. 1). It was found that the amounts of phenolics were significantly variable among different extracts (*F*-value = 35.938; *p*-value = 0.00001). It was observed that the ethyl acetate extracts of both alga and its endophyte contained the highest contents of phenolics.

Extracts were significantly different in their contents of flavonoids (*F*-value = 251.07; The *p*-value = 0.00001). The highest quantities of flavonoids (2164.7 ± 5. 5 and 1418.4 ± 7.71 µg quercetin equivalent/mg extract) were detected in the methanol extract of *C. myrica* and the ethyl acetate of *C. thiocycli* respectively.

The ethyl acetate extract of *C. myrica* was significantly contained a rich amount of saponins and tannins (778 ± 6.4 µg diosgenin equivalent/mg extract and 606 ± 1.4 µg catechol equivalent/mg extract respectively) comparing to other extracts (Fig. 1).

### Antioxidant activities

**Fig. 2:**
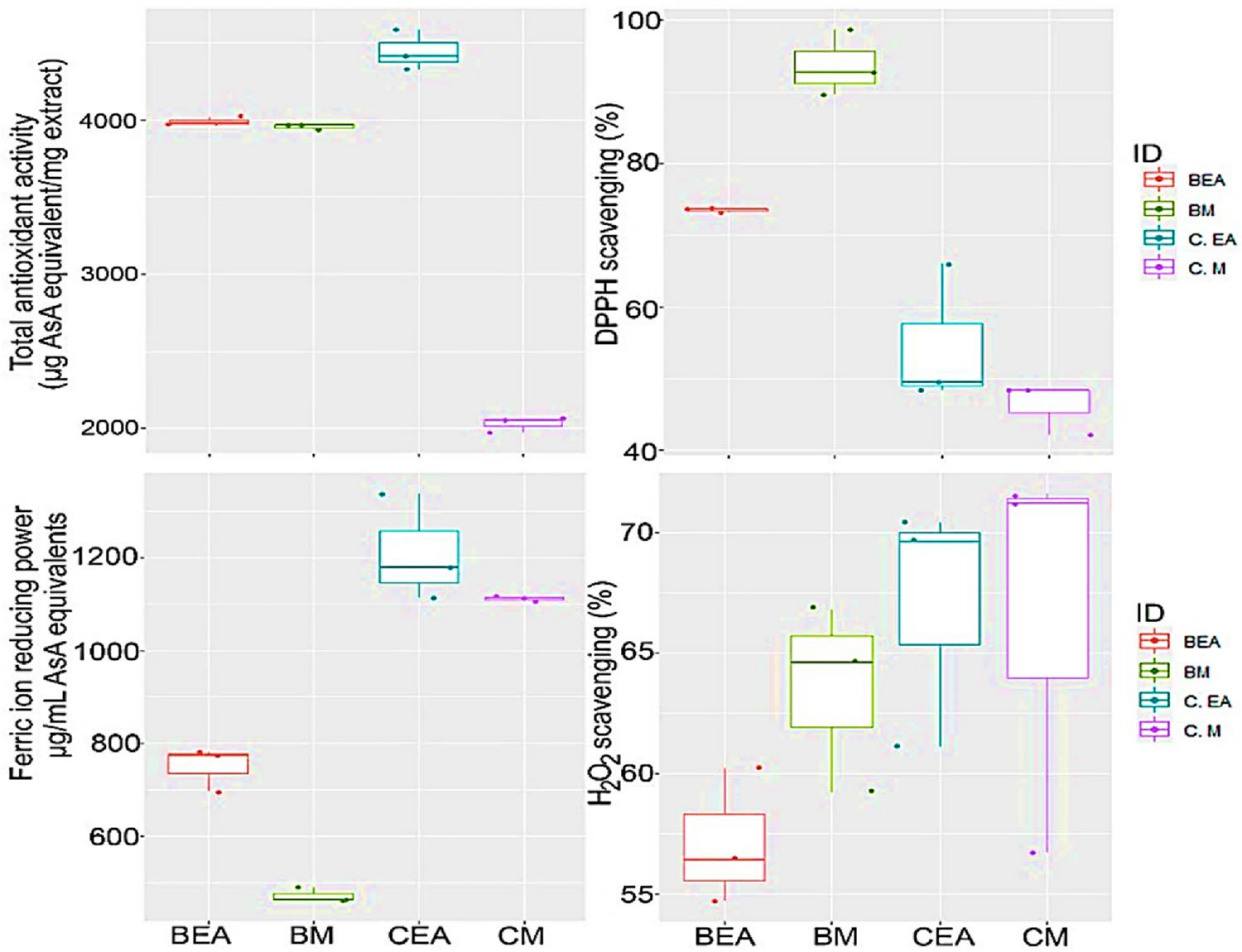
Box plot showing the differences in antioxidant activities (total antioxidant activity, DPPH scavenging activity, reducing power and H_2_O_2_ scavenging activity) among different extracts. Extracts: *BEA* *C. thiocycli* ethyl acetate, *BM* *C. thiocycli* methanol, *CEA* *C. myrica* ethyl acetate, *CM* *C. myrica* methanol

In this study, three complementary measurements i.e., total antioxidant activity, DPPH radical scavenging activity, H_2_O_2_ radical scavenging activity and Ferric ion reducing power were performed to evaluate the antioxidant activity of the different extracts (Fig. 2). Total antioxidant activity was significantly different (*F*-value = 1634.5; *p*-value = 0.00001) among different extracts (Fig. 2). Interestingly, the highest total antioxidant activities (4415.3 ± 7 and 3978.7 ± 7.1 µg ascorbic acid equivalent/mg extract) were assessed for the ethyl acetate extracts of *C. myrica* and *C. thiocycli* respectively.

The different extracts were significantly varied in their DPPH radical scavenging ability (*F*- value = 101.72; *p*-value = 0.00001). In general, the extracts of the endophyte *C. thiocycli* had higher DPPH radical scavenging activities than those of the host *C. myrica*. Among the extracts, the methanolic extract of *C. thiocycli* was the most potent with a scavenging activity percentage of 92.7%.

Scavenging potential of H_2_O_2_ radicals by the different extracts was significantly different (*F*-value = 5.1; *p*-value = 0.009). The methanolic extracts of both the host and the endophyte revealed the highest percent of scavenging activity (71.6 ± 0.3 and 66.8 ± 1.6% respectively).

The extracts displayed significant different reducing power values (*F*-value = 217.3; *p*-value = 0.00001). The extracts of the host *C. myrica* were the most potent with reducing potential ranged from 1105.5 ± 5.2 to 1338 ± 5 μg ascorbic acid equivalent/mg extract compared to those of the endophyte *C. thiocycli*.

### Anti-inflammatory activity

**Fig. 3:**
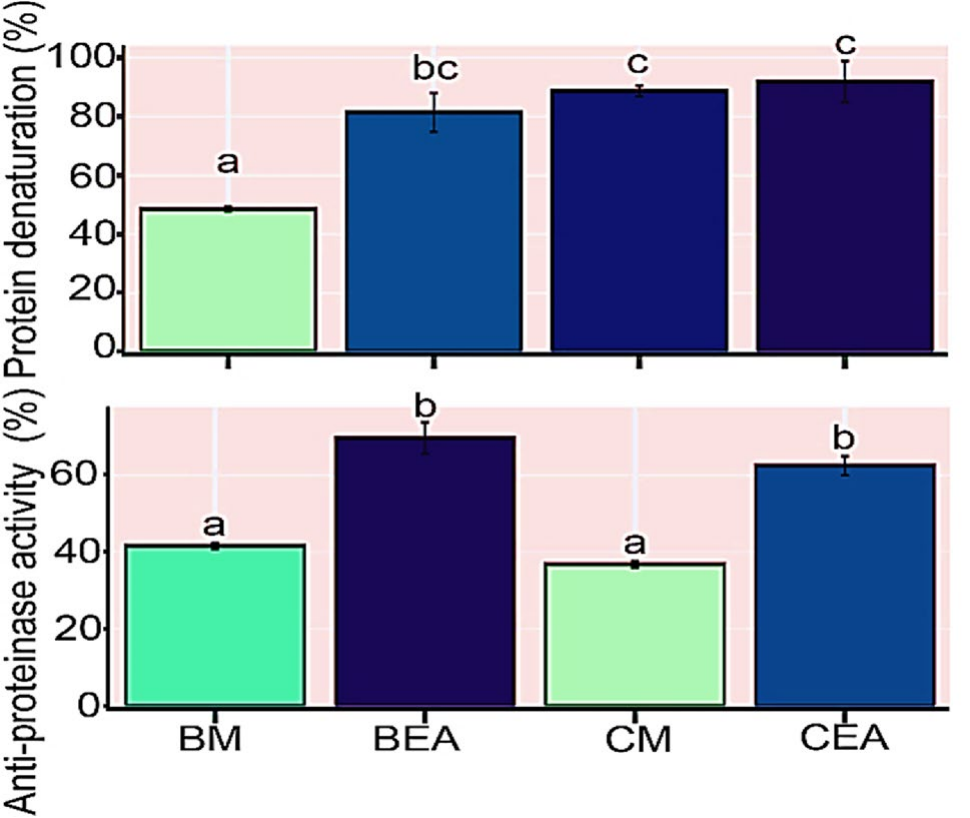
The anti-inflammatory activity of the different extracts. Extracts: *BM* *C. thiocycli* methanol, *BEA* *C. thiocycli* ethyl acetate, *CM* *C. myrica* methanol, *CEA* *C. myrica* ethyl acetate

In this study, the in vitro anti-inflammatory activity of extracts was determined by measuring their inhibitory action against protein denaturation and proteinase activity. Compared to the tested standard anti-inflammatory drug (aspirin), the highest inhibition percentage of albumin denaturation was observed in the ethyl acetate and methanolic extracts of the macroalga *C. myrica* (96.7 ± 0.5 and 89.9 ± 0.2% respectively). Whereas the anti-proteinase activity of the ethyl acetate extraction of the endophyte *C. thiocycli* was higher than those of the other tested extracts (Fig. 3).

### Antimicrobial activity

**Fig. 4:**
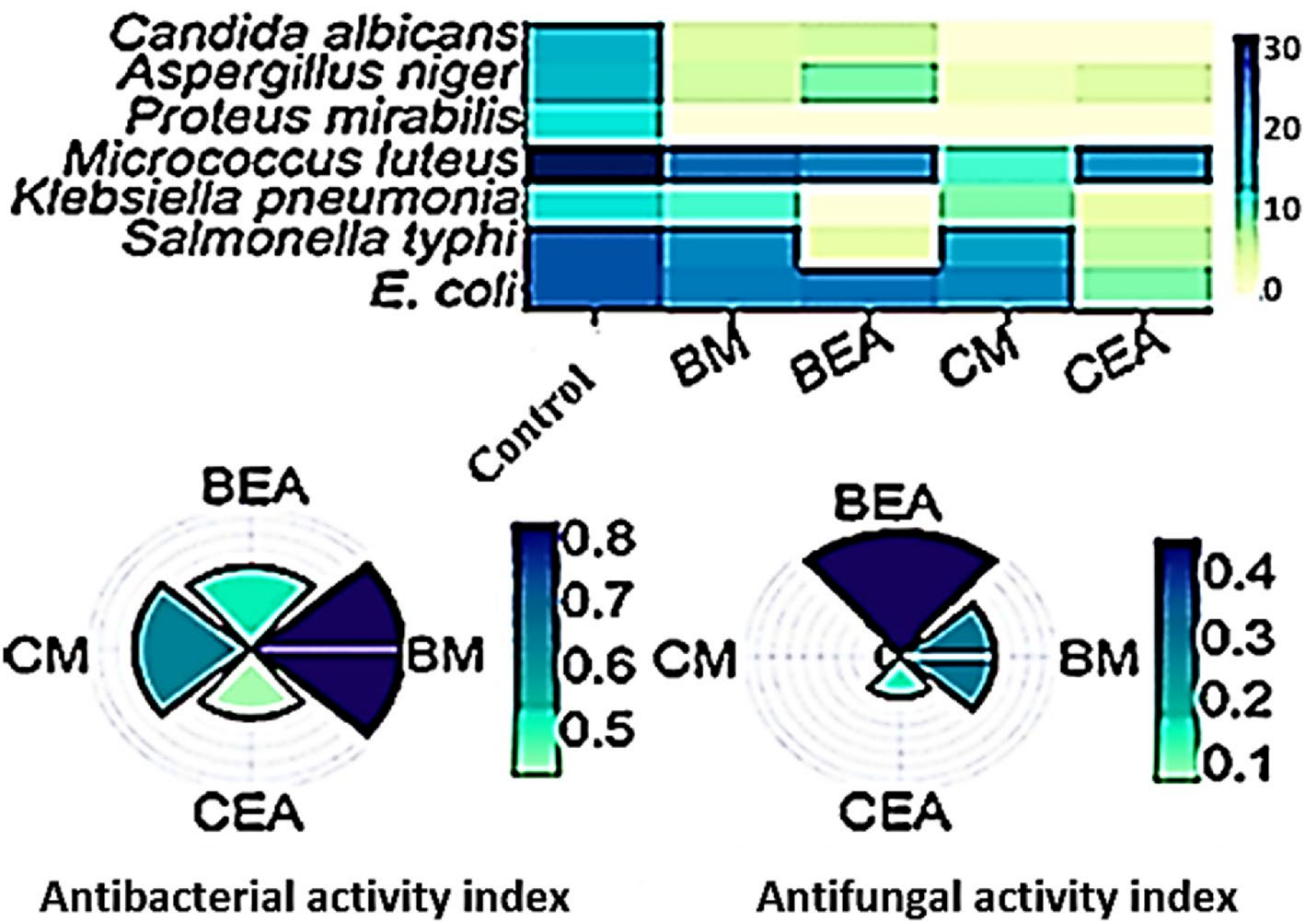
Heatmap illustrates the antimicrobial activity of the different extracts against pathogenic bacteria and fungi. Extracts: *BM* *C. thiocycli* methanol, *BEA* *C. thiocycli* ethyl acetate, *CM* *C. myrica* methanol, *CEA* *C. myrica* ethyl acetate

The antibacterial and antifungal potential of extracts against *Escherichia coli, Salmonella typhi, Klebsiella pneumoniae, Micrococcus luteus, Proteus mirabilis, Aspergillus niger* and *Candida albicans* was evaluated (Fig. 4). The different extracts had significant different effects on the tested bacteria (*F*-value = 7.07; *p*-value = 0.001). Interestingly, the methanolic extracts of both *C. thiocycli* and *C. myrica* showed the broadest antibacterial spectrum with activity index values of 0.81 ± 0.021 and 0.62 ± 0.035 respectively (Fig. 4). On the other hand, the ethyl acetate extract of *C. thiocycli* exhibited the most potent activity against the tested fungi with activity index value of 0.46 ± 0.028 (Fig. 4).

**Fig. 5:**
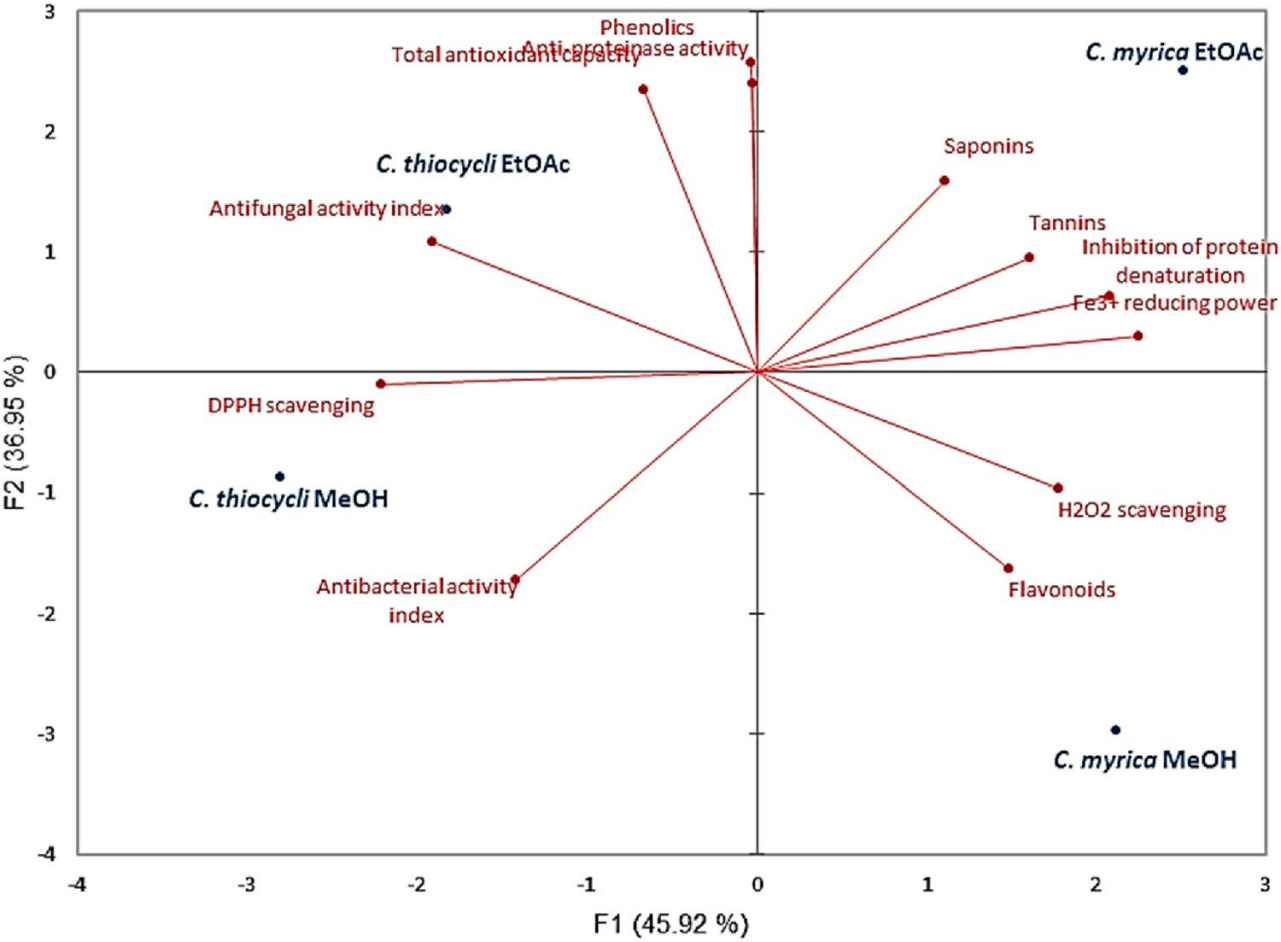
Principal component analysis (PCA) distinguishes between bioactive secondary metabolites in the different extracts. The red arrows radiating from the center of the plot represent the loading values for variables (contents of metabolites and their bioactivities), showing the differences in the direction (angle) and magnitude (length) between the different extracts. Extracts: *EtOAc* ethyl acetate, *MeOH* methanol

PCA was used to confirm these results. Interestingly, extracts were categorized into four groups (Fig. 5). The first group located in the upper left side of the plot included the ethyl acetate extract of *C. thiocycli* which contained the highest content of phenolics and was higher in total antioxidant, anti-proteinase, and antifungal activities than the other extracts. The second group included the ethyl acetate extract of *C. myrica* which was characterized by high contents of saponins and tannins and exhibited high percentage of protein denaturation inhibition and Fe^3+^ reducing power (aligned in the upper right side of the plot). The third group was aligned in the lower left side of the plot and included the methanolic extract of *C. thiocycli* which exhibited high DPPH scavenging and antibacterial activities. The fourth group at the lower right side of the plot contained the methanolic extract of *C. myrica* which characterized by high content of flavonoids and revealed higher H_2_O_2_ scavenging ability.

### GC–MS analysis of extracts

In the present study, The GC–MS analysis of the ethyl acetate and methanolic crude extracts of *C. myrica* and *C. thiocycli* showed a mixture of various compounds. Seventeen peaks were detected for ethyl acetate extracts of both *C. myrica* and *C. thiocycli*, while the total number of the main peaks that were observed for methanolic extracts of both *C. myrica* and *C. thiocycli* were twenty-five. The major bioactive compounds that were characterized and identified in the ethyl acetate and methanolic extracts of *C. myrica* and *C. thiocycli* were shown in (Tables 1, 2).

**Table 1:** The major bioactive compounds identified in the ethyl acetate extracts of the macroalga *C. myrica* and its associated bacterial endophyte *C. thiocycli* by GC–MS analysis

Extract	Compounds	Retention time (min)	Molecular weight	Peak area (%)	Bioactivity as per literatures	Reference
*C. myrica*	Dibutyl phthalate (C_16_H_22_O_4_)	15.246	278	13.611	Antimicrobial	Roy and Laskar (2006)
	Heptadecane (C_17_H_36_)	9.089	240	11.097	Anti-inflammatory	Kim et al. (2013)
	Octacosane (C_28_H_58_)	11.378	394	9.953	antimicrobial, antioxidant, anti-inflammatory	Khatua et al. (2016)
	Tetracosane (C_24_H_50_)	9.496	338	8.969	Antioxidant	Paudel et al. (2019)
	Pentacosane (C_25_H_52_)	11.939	352	6.829	Antimicrobial	Marrufo et al. (2013)
*C. thiocycli*	Dodecane, 2,6,11-trimethyl- (C_15_H_32_)	9.089	212	12.991	Antibacterial	Rahbar et al. (2012)
	Dibutyl phthalate (C_16_H_22_O_4_)	15.241	278	11.619	Antimicrobial	Roy and Laskar (2006)
	Dodecane (C_12_H_26_)	9.496	170	11.388	Antibacterial	Padma et al. (2019)
	2-Bromo dodecane (C_12_H_25_Br)	11.378	248	10.371	Antibacterial	Aagboke and Aftama (2016)
	Octacosane (C_28_H_58_)	11.939	394	7.065	Antimicrobial, antioxidant, anti-inflammatory	Khatua et al. (2016)

**Table 2:** The major bioactive compounds identified in the methanolic extracts of the macroalga *C. myrica* and its associated bacterial endophyte *C. thiocycli* by GC–MS analysis

Extract	Compounds	Retention time (min)	Molecular weight	Peak area (%)	Bioactivity as per literatures	Reference
*C. myrica*	Heneicosane (C_21_H_44_)	9.089	296	12.250	Antimicrobial	Vanitha et al. (2020)
	Eicosane (C_20_H_42_)	9.495	282	11.421	Anti-inflammatory	Okechukwu (2020)
	Pentadecane (C_15_H_32_)	11.378	212	9.493	Antimicrobial	Barretto and Vootla (2018)
	Pentacosane (C_25_H_52_)	11.939	352	7.693	Antimicrobial	Marrufo et al. (2013)
	Octacosane (C_28_H_58_)	15.240	394	5.750	antimicrobial, antioxidant, anti-inflammatory	Khatua et al. (2016)
*C thiocycli*	Eicosane (C_20_H_42_)	9.089	282	12.393	Anti-inflammatory	Okechukwu (2020)
	Tetracosane (C_24_H_50_)	9.496	338	11.261	Antioxidant	Paudel et al. (2019)
	Heptacosane (C_27_H_56_)	11.384	380	11.152	Antibacterial	Duke (1992)
	Hentriacontane (C_31_H_64_)	11.939	436	9.048	Anti-inflammatory	Kim et al. (2011)
	2-methyloctacosane (C_29_H_60_)	14.531	408	6.899	Antimicrobial	Barretto and Vootla (2018)

## Discussion

Algae are the main primary producers that represent the key elements of the aquatic environment (Harder 2009). Bacteria are naturally inhabiting the environments of micro- and macroalgae, and they are dominant among the primary colonizers that associate with algal surfaces and tissues (Lachnit et al. 2011). Until now, there are many gaps in our understanding of the relationships and possible specific associations in aquatic environments. Although some studies have been recently concerned the interactions between marine organisms, there are insufficient investigations on macroalgae–bacteria interactions (Goecke et al. 2010).

In recent years, it has been reported that secondary metabolites particularly play a significant role in regulating the macroalgae–bacteria relationships in marine ecosystems (Sneed and Pohnert 2011a). In the present study, the secondary metabolites produced by the marine brown macroalga *Cystoseira myrica* and its associated bacterial endophyte *Catenococcus thiocycli* strain QCm, and their biological activities were investigated.

**Fig. 6:**
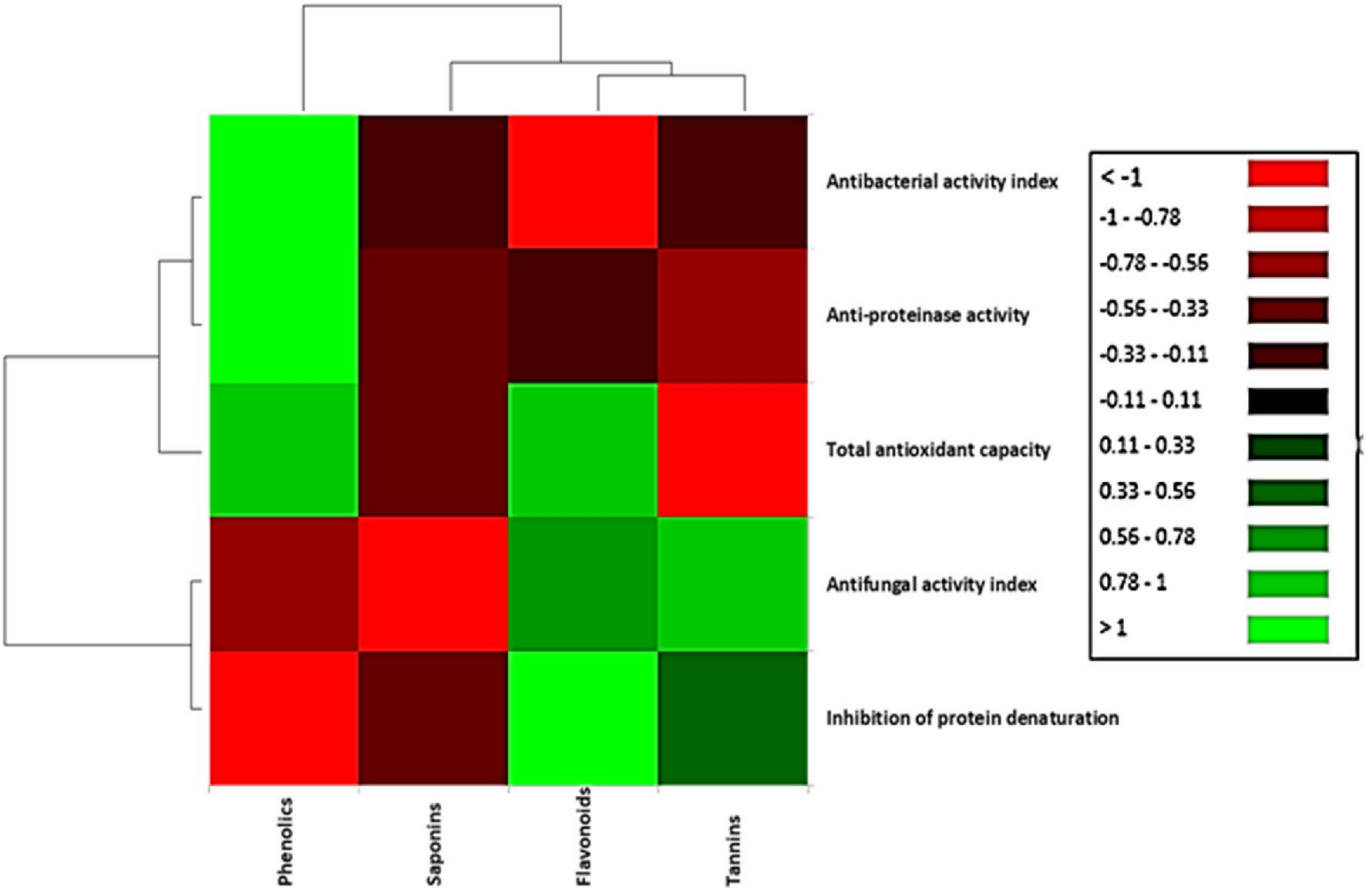
Heatmap showing Pearson’s correlation analysis (correlation coefficient *r*) among the contents of secondary metabolites and the biological activities of the different extracts

Interestingly, it was found that *C. myrica* and associated *C. thiocycli* produce similar array of secondary metabolites including phenolics, flavonoids, saponins and tannins (Fig. 1). In the current study, the total antioxidant activity of the extracts was positively correlated with their contents of phenolics and flavonoids (Fig. 6). This agreed with the findings of the others who reported the presence of positive correlations between phenolic contents and antioxidant activities (Wang et al. 2010; Contreras-Calderón et al. 2011; Baharfar et al. 2015). Furthermore, linear proportional between total flavonoids and antioxidant activity was previously reported (Jayaprakasha et al. 2004; Ghasemzadeh et al. 2012).

During inflammatory reactions, proteinases significantly involve in tissue damage, so proteinase inhibitors like phenolics and flavonoids can provide a high level of protection (Leelaprakash and Mohan-Dass 2011). In the present study, the anti-inflammatory activity of the different extracts has been positively correlated with their phenolic and flavonoid contents (Fig. 6). This was in accordance with the findings of Diaz et al. (2012) and Naz et al. (2017) who reported the positive correlation between the levels of phenolic and flavonoid compounds in the extracts and their anti-inflammatory activities. Hence, the bioactive properties of the present extracts attributed to their high contents of phenolics and flavonoids.

On the other hand, there was a positive correlation between phenolic contents and antibacterial activity of the extracts (Fig. 6). This may attribute to the inhibitory effect of phenolics on nucleic acid biosynthesis and metabolic processes (Babaa and Malikb 2014; Naz et al. 2017). While antifungal activity of the extracts was attributed to flavonoids and tannins. It was previously reported that flavonoids effect on proteins and enzymes of the fungal cells and alter their configuration and activity (Kanwal et al. 2010), and tannins disrupt cell wall and plasma membrane (Zhu et al. 2019).

GC–MS analysis of *C. myrica* extracts revealed the presence of 17 constituents in the ethyl acetate extract and 25 constituents in the methanolic extract. Based on the peak area and retention time, the major identified compounds that were found in high amounts were dibutyl phthalate, heptadecane, octacosane, tetracosane, pentacosane, heneicosane, eicosane and pentadecane (Tables 1, 2). These chemical compounds have been reported to have antioxidant, antimicrobial, and anti-inflammatory potential (Roy and Laskar 2006; Kim et al. 2013; Marrufo et al. 2013; Khatua et al. 2016; Barretto and Vootla 2018; Paudel et al. 2019; Okechukwu 2020; Vanitha et al. 2020). On the other hand, 17 and 25 chemical compounds were identified in the ethyl acetate and methanolic extracts of *C. thiocycli* respectively (Tables 1, 2). The major peaks displayed by GC–MS chromatogram of the ethyl acetate extract were for dodecane, 2,6,11-trimethyl-, dibutyl phthalate, dodecane, 2-Bromo dodecane and octacosane compounds. While the major compounds detected in the methanolic extract were eicosane, tetracosane, heptacosane, hentriacontane and 2-methyloctacosane. Antioxidant, antimicrobial, and anti-inflammatory activity of these compounds were well previously documented (Duke 1992; Roy and Laskar 2006; Kim et al. 2011; Rahbar et al. 2012; Aagboke and Aftama 2016; Khatua et al. 2016; Barretto and Vootla 2018; Padma et al. 2019; Paudel et al. 2019; Okechukwu 2020).

## Conclusion

The present study was carried out to contribute towards our understanding of relationships between macroalgae and their associated endophytic bacteria regarding the production of bioactive secondary metabolites. The output results of the study revealed that the brown marine macroalga *C. myrica* and associated bacterial endophyte *C. thiocycli* strain QCm produce similar array of bioactive secondary metabolites which possess significant antioxidant, anti-inflammatory, and antimicrobial activities. This study suggests that *C. myrica* and associated *C. thiocycli* are promising source for pharmaceutical agents. Therefore, we recommend the separation and purification of active compounds and evaluating their activities using in vivo studies.

## References

[CR1] Aagboke A, Aftama AA (2016). Bioactive components and antibacterial activities of n-hexane extract of *Moringa oleifera* root bark on clinical isolates of methicilin resistant *Staphylococcus aureus*. Int J Curr Res Chem Pharm sci.

[CR2] Aleem AA (1993). The marine algae of Alexandria.

[CR3] Ameen F, AlNadhari S, Al-Homaidan AA (2021). Marine microorganisms as an untapped source of bioactive compounds. Saudi J Biol Sci.

[CR4] Babaa SA, Malikb SA (2014). Evaluation of antioxidant and antibacterial activity of methanolic extracts of *Gentiana kurroo* royle. Saudi J Biol Sci.

[CR5] Baharfar R, Rahmani Z, Mohseni M, Azimi R (2015). Evaluation of the antioxidant and antibacterial properties of ethanol extracts from berries, leaves and stems of Hedera pastuchovii Woron. ex Grossh. Nat Prod Res.

[CR6] Barretto DA, Vootla SK (2018). GC-MS analysis of bioactive compounds and antimicrobial activity of *cryptococcus rajasthanensis* ky627764 isolated from *bombyx mori* gut microflora. Int j adv Res.

[CR7] Brand-Williams W, Cuvelier ME, Berset C (1995). Use of a free radical method to evaluate antioxidant activity. LWT Food Sci Technol.

[CR8] Clinical and Laboratory Standards Institute (2010) Performance standards for antimicrobial susceptibility testing: nineteenth informational supplement. 19 ed., 29(3). Wayne, PA: Clinical and Laboratory Standards Institute; p. 149. M100-S19.

[CR9] Contreras-Calderón J, Calderón-Jaimes L, Guerra-Hernández E, García-Villanova B (2011). Antioxidant capacity, phenolic content and vitamin C in pulp, peel and seed from 24 exotic fruits from Colombia. Food Res Int.

[CR10] Diaz P, Jeong SC, Lee S, Khoo C, Koyyalamudi SR (2012). Antioxidant and anti-inflammatory activities of selected medicinal plants and fungi containing phenolic and flavonoid compounds. Chin Med.

[CR11] Duke JA (1992). Handbook of phytochemical constituents of GRAS herbs and other economic plants.

[CR12] Erbabley NYGF, Junianto J (2020). Chemical characteristics and phytochemicals of the brown alga *Sargassum filipendulla* from kelanit waters of southeast Maluku. Egypt J Aquat Biol Fish.

[CR13] Ganesan P, Kumar CSBN (2008). Antioxidant properties of methanol extract and its solvent fractions obtained from selected Indian red seaweeds. Bioresour Technol.

[CR14] Ghasemzadeh A, Azarifar M, Soroodi O, Jaafar HZE (2012). Flavonoid compounds and their antioxidant activity in extract of some tropical plants. J Med Plants Res.

[CR15] Goecke F, Labes A, Wiese J, Imhoff JF (2010). Chemical interactions between marine macroalgae and bacteria. Mar Ecol Prog Ser.

[CR16] Harder T, Flemming HC, Murthy PS, Venkatesan R, Cooksey K (2009). Marine epibiosis: concepts, ecological consequences, and host defence. Marine and industrial biofouling.

[CR17] Jayaprakasha GK, Jaganmohan Rao L, Sakariah KK (2004). Antioxidant activities of flavonoid in different in vitro model systems. Bioorg Med Chem.

[CR18] Kanwal Q, Hussain I, Latif Siddiqui H, Javaid A (2010). Antifungal activity of flavonoids isolated from mango (Mangifera indica L.) leaves. Nat Prod Res.

[CR19] Khatua S, Pandey A, Biswas SJ (2016). Phytochemical evaluation and antimicrobial properties of *Trichosanthes dioica* root extract. J Pharmacogn Phytochem.

[CR20] Kim S-J, Chung W-S, Kim S-S, Ko S-G, Um J-Y (2011). Anti-inflammatory Effect of *Oldenlandia diffusa* and its constituent, hentriacontane, through suppression of caspase-1 activation in mouse peritoneal macrophages. Phytother Res.

[CR21] Kim DH, Park MH, Choi YJ, Chung KW (2013). Molecular study of dietary heptadecane for the anti-inflammatory modulation of NF-kB in the aged kidney. PLoS ONE.

[CR22] Lachnit T, Meske D, Wahl M, Harder T, Schmitz R (2011). Epibacterial community patterns on marine macroalgae are host-specific but temporally variable. Environ Microbiol.

[CR23] Leelaprakash G, Mohan-Dass S (2011). In vitro anti-inflammatory activity of methanol extract of *Enicostemma axillare*. Int J Drug Dev Res.

[CR24] Madkour FF, El-Shoubaky GA, Ebada MA (2019). Antibacterial activity of some seaweeds from the red seacoast of Egypt. Egypt J Aquat Biol Fish.

[CR25] Marrufo T, Nazzaro F, Mancini E, Fratianni F (2013). Chemical composition and biological activity of the essential oil from leaves of Moringa oleifera Lam. cultivated in Mozambique. Molecules.

[CR26] Marzoqi A, Hameed I, Salah I (2015). Analysis of bioactive chemical components of two medicinal plants (*Coriandrum sativum* and *Melia azedarach*) leaves using gas chromatography-mass spectrometry (GC-MS). African J Biotechnol.

[CR27] Mizushima Y, Kobayashi M (1968). Interaction of anti-inflammatory drugs with serum proteins, especially with some biologically active proteins. J Pharm Pharmacol.

[CR28] Naz R, Ayub H, Nawaz S (2017). Antimicrobial activity, toxicity, and anti-inflammatory potential of methanolic extracts of four ethnomedicinal plant species from Punjab. Pakistan BMC Complement Altern Med.

[CR29] Noha AM, Hossam MH, Mostafa ER (2013). Diketopiperazine derivatives from *Enterobacter cloacae* isolated from the red sea alga *Cystoseira myrica*. Egypt Pharmaceut J.

[CR30] Nurjanah NM, Anwar E, Luthfiyana N, Hidayat T (2017). Identification of bioactive compounds of seaweed sargassum sp. and eucheuma cottonii doty as a raw sunscreen cream. B. Life Environ Sci.

[CR31] Okechukwu PN (2020). Evaluation of anti-inflammatory, analgesic, antipyretic effect of eicosane, pentadecane, octacosane, and heneicosane. Asian J Pharm Clin Res.

[CR32] Oyedapo OO, Famurewa AJ (2008). Antiprotease and membrane stabilizing activities of extracts of *fagara zanthoxyloides*, *olax subscorpioides* and *tetrapleura tetraptera*. Int J Pharmacogn.

[CR33] Padma M, Ganesan S, Jayaseelan T, Azhagumadhavan S (2019). Phytochemical screening and GC–MS analysis of bioactive compounds present in ethanolic leaves extract of Silybum marianum (L.). J Drug Deliv Ther.

[CR34] Paudel MR, Chand MB, Pant B, Pant B (2019). Assessment of antioxidant and cytotoxic activities of extracts of *Dendrobium crepidatum*. Biomolecules.

[CR35] Prieto P, Pineda M, Aguilar M (1999). Spectrophotometric quantitation of antioxidant capacity through the formation of a phosphomolybdenum complex: specific application to the determination of vitamin E. Anal Biochem.

[CR36] Rahbar N, Shafagha A, Salimi F (2012). Antimicrobial activity and constituents of the hexane extracts from leaf and stem of *Origanum vulgare* L. sp. Viride (Boiss.) Hayek. Growing wild in Northwest Iran. J Med Plants Res.

[CR37] Rashad S, El-Chaghaby GA (2020). Marine Algae in Egypt: distribution, phytochemical composition, and biological uses as bioactive resources (a review). Egypt J Aquat Biol Fish.

[CR38] Roy RN, Laskar S, Sen SK (2006). Dibutyl phthalate, the bioactive compound produced by streptomyces albidoflavus 321.2. Microbiol Res.

[CR39] Ruch RJ, Cheng SJ, Klaunig JE (1989). Prevention of cytotoxicity and inhibition of intercellular communication by antioxidant catechins isolated from Chinese green tea. Carcinogenesis.

[CR40] Singh B, Sahu PM, Sharma MK (2002). Anti-inflammatory and antimicrobial activities of triterpenoids from *Strobilanthes callosus* Nees. Phytomedicine.

[CR41] Sneed JM, Pohnert G (2011). The green alga *Dictyosphaeria ocellata* and its organic extracts alter natural bacterial biofilms communities. Biofouling.

[CR42] Soria-Mercado IE, Villarreal-Gómez LJ, Rivas GG, Sánchez NEA (2012) Bioactive compounds from bacteria associated to marine algae. In: Sammour R (ed) Biotechnology - molecular studies and novel applications for improved quality of human life. InTech. http://www.intechopen.com/books/biotechnology-molecular-studies-and-novel-applications-for-improved-quality-of-human-life/bioactive-compounds-from-bacteria-associated-to-marine-algae. Accessed 14 Dec 2017

[CR43] Vanitha V, Vijayakumar S, Nilavukkarasi M (2020). Heneicosane- a novel microbicidal bioactive alkane identified from *Plumbago zeylanica* L. Ind Crop Prod.

[CR44] Visweswari G, Christopher R, Rajendra W (2013). Phytochemical screening of active secondary metabolites present in *Withania somnifera* root: role in traditional medicine. IJPSR.

[CR45] Wang H, Gan D, Zhang X, Pan Y (2010). Antioxidant capacity of the extracts from pulp of *Osmanthus fragrans* and its components. Lebenson Wiss Technol.

[CR46] Yen GC, Chen HY (1995). Antioxidant activity of various tea extracts in relation to their antimutagenicity. J Agric Food Chem.

[CR47] Zhu C, Lei M, Andargie M, Zeng J, Li J (2019). Antifungal activity and mechanism of action of tannic acid against *Penicillium digitatum*. Physiol Mol Plant Pathol.

